# Prevalence, Virulence, and Antibiotics Gene Profiles in *Lactococcus garvieae* Isolated from Cows with Clinical Mastitis in China

**DOI:** 10.3390/microorganisms11020379

**Published:** 2023-02-02

**Authors:** Xinmei Xie, Zihao Pan, Yong Yu, Lirong Yu, Fan Wu, Jing Dong, Tiancheng Wang, Lin Li

**Affiliations:** 1 College of Animal Science & Veterinary Medicine, Shenyang Agricultural University, Shenyang 110866, China; 2 College of Veterinary Medicine, Nanjing Agricultural University, Nanjing 210095, China

**Keywords:** antimicrobial resistance, bovine clinical mastitis, *Lactococcus garvieae*, pathogenicity, virulence gene

## Abstract

*Lactococcus garvieae* (*L. garvieae*) is a pathogenic gram-positive, catalase-negative (GPCN) bacterium that causes bovine mastitis. A total of 49 *L. garvieae* isolates were identified from 1441 clinical mastitis (CM) samples. The pathogenic effects of *L. garvieae* were studied with two infection models: bovine mammary epithelial cells cultured in vitro and murine mammary infections in vivo. The overall farm prevalence was 15.5% (13/84 farms in 9/19 provinces) and sample prevalence was 3.40% (49/1441). Post-treatment somatic cell count (SCC) post *L. garvieae* infection was significantly higher than the other GPCN pathogens isolated, and the bacteriological cure fraction was 41.94% (13/31) after intramammary antibiotic treatment. All *L. garvieae* isolates were resistant to rifaximin, 12.24% of isolates were resistant to cephalexin, and 10.20% (5/49) were multidrug-resistant (MDR). The most prevalent virulence genes were Hemolysin 1 (hly1)(100%), Hemolysin 2 (hly2) (97.96%), NADH oxidase (NADHO) (100%), Superoxide dismutase (SOD) (100%), Adhesin Pav (Pav) (100%), Adhesin PsaA (PsaA) (100%), Enolase (eno) (100%), Adhesin cluster 1(AC1) (100%), Adhesin cluster 2 (AC2) (100%), and several exopolysaccharides. *L. garvieae* rapidly adhered to bovine mammary epithelial cells, resulting in an elevated lactate dehydrogenase release. Edema and congestion were observed in challenged murine mammary glands and bacteria were consistently isolated at 12, 24, 48, 72, and 120 h after infection. We concluded that *L. garvieae* had good adaptive ability in the bovine and murine mammary cells and tissue. Given the resistance profile, penicillin and ampicillin are potential treatments for CM cases caused by *L. garvieae*.

## 1. Introduction

Mastitis, an inflammatory process of the mammary gland, is the most common bacterial disease [1] and one of the most costly diseases of dairy cattle [2]. *Lactococcus* species have been known to be associated with mastitis since as early as 1932 [3]. However, GPCN streptococci or streptococci-like bacteria including *Streptococcus*, *Enterococcus*, *Aerococcus*, and *Lactococcus* are phenotypically and biochemically alike. This means the identification of *Lactococcus* species has been inaccurate and unreliable in many studies and diagnostic laboratories [4]. The incidence of *Lactococcus* species identified on-farm may have been historically underreported or was phenotypically identified as *Streptococcus uberis (S. uberis)* or *Streptococcus* spp. [5]. Thus, little is known about the clinical importance of this genus as a mastitis pathogen, and awareness and focus have only increased in recent years.

Based on phenotypic similarities, *Lactococcus* species were initially assigned to the genus *Streptococcus*, and a new genus was assigned to *Lactococcus* in 1985 [6]. *L. garvieae* was known as an emergent disease affecting many fish species and it is considered a potential zoonotic microorganism [7]. This is because it is known to cause several opportunistic human infections, such as endocarditis [8], diverticulitis [9], peritonitis [9], infective spondylodiscitis [10], liver abscess [11], urinary infection [12], subdural hematoma [13], sepsis [14,15,16], bacteremia [9], and late onset periprosthetic infection of the hip [17]. More recently, *L. garvieae* has been reported in sepsis cases in human patients receiving platelet concentrates from the same donor, one day after transfusion [16]. This highlights the importance of surveillance of this emerging pathogen for humans, especially those who have predisposing health conditions [10]. *L. garvieae* has also been identified in other animal species (e.g., pigs with pneumonia [18], canine tonsils, sheep, goats, cats, horses, camels, turtles, snakes, and crocodiles [19]); it has also been found in environmental sources, water [20], sand [21], soil [22], and in foodstuffs including radish, broccoli [23], fennel, celery, broccoli, zucchini, wheat flour, and bran [24].

In cattle, *L. garvieae* was first identified from a mastitis case in 1983 [25], and it was then found in buffalo and cows in Spain [20,26]. Reports from a variety of different countries include clinical and subclinical mastitis cases, such as in dairy cows in Belgium [3], in raw cow’s milk [27,28], cheese made from cow’s milk [29,30], fermented cow’s milk [31], and in beef [32]. In 2016, a *Lactococcus* genus mastitis outbreak was reported in the USA [33]. 

Hemolysis-, adhesion-, and immune-evasion-related and other putative virulence genes of *L. garvieae* have been described and detected, and may play an important role in bacterial virulence [34]. Hemolysins are proteins and lipids that cause cell membrane damage during infection. Many bacterial pathogens are capable of expressing different adhesins, and different adhesins are expressed at different stages during infection, and their synergistic effect plays an important role in the pathogenicity of pathogenic bacteria [35]. LPxTG is a surface protein that covalently binds peptidoglycans isolated from many gram-positive bacteria including *L. garvieae*, and has an adhesive effect [36]. The formation of a capsule improved the resistance of pathogenic bacteria to phagocytosis in fish-derived *L. garvieae* [37]. The polysaccharide capsule of *L. garvieae* has been widely described as a major virulence factor involved mainly in the evasion of the host’s immune response [7]. *NADHO* and *SOD* are enzymes that aid in the survival of pathogens in aerobic environments; the bacteria protect themselves by producing these enzymes to avoid being killed [34]. Different pathogenic *L. garvieae* isolates have different phenotypes; thus, it is important to investigate the putative virulence genes of *L. garvieae* strains [34].

Multidrug resistant pathogens are defined as being resistant to three or more classes of antimicrobial agents [38]. The widespread use of antibiotics in agriculture, such as for the treatment and prevention of infections, has led to the selection of drug-resistant and multidrug-resistant bacteria [39]. Monitoring multidrug-resistance in pathogenic bacteria is important because drug-resistant bacterial infections reduce treatment options, increase health care costs, and may lead to increased morbidity and mortality [40]. 

CM samples (n = 1441) were taken from commercial Chinese dairy farms and 49 isolates were ultimately identified as *L. garvieae*. This was the first time the emerging mastitis pathogen has been detected in Chinese CM samples. There is little research on the characterization of *L. garvieae* from dairy cattle mastitis. Therefore, the objectives of this study were to determine the epidemiology, SCC resolution and bacteriological cure fraction, antimicrobial resistance profile, detection of putative virulence genes, and pathogenic effects of *L. garvieae* isolated from CM samples with MAC-T cell and murine mammary infection models.

## 2. Materials and Methods

### 2.1. Statement of Ethics

The present study was conducted according to the ethical guidelines of Shenyang Agricultural University. Prior to commencement, the animal study was reviewed and approved by the Laboratory Animal Management Committee of Shenyang Agricultural University (protocol: 2021060102).

### 2.2. Dairy Farm Information and Clinical Mastitis Sample Collection

CM milk samples (n = 1441) were obtained from 84 Chinese commercial dairy farms from 19 different provinces. Abnormal milk (e.g., clots, flake, and watery milk) was identified by farm personnel as CM samples. CM samples were aseptically collected from individual quarters by the authors of this study or trained on-farm personnel. The CM samples for each farm were collected within a 7 day time span. The samples were quickly frozen (−20 °C) overnight and then shipped to Shenyang Agricultural University in Shenyang, China for further identification. The CM sample collection period was from March 2020 to July 2021.

### 2.3. Bacterial Culture of L. garvieae

Sheep blood agar and brain heart infusion (BHI) agar were used to isolate and purify the colony of the GPCN cocci growing on sheep blood agar from 1441 CM milk samples. Then, BHI broth was used to proliferate bacteria from the colonies that grew on the agar. Gram staining and scanning electron microscopy (SEM) were carried out in order to observe the morphology of *L. garvieae.*

### 2.4. 16 S rDNA Sequencing Identification and Biochemical Testing

Bacterial DNA was extracted from 248 isolates using a bacterial DNA extraction kit (Sangon Biotech (Shanghai) Co., Ltd., Shanghai, China) according to the manufacturer’s instructions. The extracted DNA was used as a PCR template for amplification; GPCN streptococci-like isolates were determined by 16S rDNA sequencing [41], where primer p27f (5′-AGAGTTTGATCCTGGCTCAG-3′) and primer 1492r (5′-TACGGCTACCTTGTTACGACTT-3′) were used to amplify a 1460-bp product of the 16S rDNA gene. The PCR cycling conditions included an initial denaturation step at 95 °C for 3 min, followed by 35 cycles at 95 °C for 15 s, 55 °C for 15 s, and 72 °C for 1 min, with a final extension step at 72 °C for 5 min. The PCR products were subjected to sequencing (Sanger sequencing by Sangon Biotech (Shanghai) Co., Ltd., Shanghai, China) after verification on 1.2% agarose gel. The 16S rDNA sequences were compared with sequences deposited in the nucleotide database of the National Center for Biotechnology Information. Identification was deemed reliable if the values for sequence similarities were ≥99%. The biochemical reacting kit (Qingdao Hi-Tech Industrial Park Haibo Biotechnology Co., Ltd., Qingdao, China) was used for biochemical testing. A total of 11 reagents (ribose, sucrose, lactose, liquid gelatin, sorbitol, maltose, esculin, galactose, VP, trehalose, and glucose) were fermented with isolates, following the manufacturer’s instructions. Briefly, isolates were seeded in the tubes that were subsequently cultured in an incubator at 37 °C for the required time; some of the reagents needed further operations and the colors were compared with negative tubes.

### 2.5. Post-Treatment Milk Sample Collection for SCC and Bacteriological Cure Evaluation

The post-intramammary-antibiotic-treatment milk samples were collected from the farm with the highest prevalence of *L. garvieae*. The dairy farm, located in northwest China, had an average of 6400 milking cows during the study period. Cows were fed a TMR and housed in freestall barns with sand bedding. Lactating cows were milked thrice daily in two rotary parlors. Milk samples were aseptically collected from the same individual quarter first identified as CM 17 ± 3 d after an extended therapy of 5 d of antimicrobial intramammary treatment (Ubrolexin, Boehringer Ingelheim, Ingelheim am Rhein, Germany) instead of the standard 2 d regimen, and with anti-inflammatory treatment (meloxicam, Boehringer Ingelheim, Germany) for bacteriological cure evaluation. A bacteriological cure was defined as being *L. garvieae* culture-positive in the clinical mastitis sample, and culture-negative in the post-treatment milk sample. After collection, the quarter milk sample was shaken several times to ensure good mixing and then tested for SCC with the DeLaval DCC instrument (Delaval (Tianjian) Co., Ltd., Tianjin, China).

### 2.6. Growth Curve of Lactococcus garvieae

The growth curves of one isolate of *L. garvieae* (LG41), one isolate of *L. lactis*, one strain of *Staphylococcus aureus* (*S. aureus*), and one isolate of *Enterococcus faecalis* (*E. faecalis*) were assessed simultaneously. The *S. aureus* was ATCC 29213, and the remaining isolates from CM milk samples from the same dairy farm had the highest *L. garvieae* prevalence. The mediums were prepared according to the manufacturer’s instructions. For each isolate, 30 μL of the bacterial solution was added to 3 mL BHI in 5 mL sterile tubes for each different isolate, and they were placed on a constant temperature shaker (37 °C, 220 rpm). At 0, 0.5, 1, 1.5, 2, 2.5, 3, 3.5, 4, 6, 8, 10, 12, 14, 16, 18, 20, 22, and 24 h, the optical density (OD) of each bacterial suspension was determined using 3 tubes per isolate at 600 nm in a UV spectrophotometer (Shimadzu Corporation, Kyoto, Japan). Each experiment was performed in triplicate.

### 2.7. Antimicrobial Resistance Determination

The minimal inhibitory concentrations (MIC) for penicillin (β-lactams), cephalexin (β-lactams), ampicillin (β-lactams), ceftiofur (β-lactams), cefquinome (β-lactams), lincomycin (lincosamide class), oxytetracycline (Tetracycline class), marbofloxacin (Quinolone class), rifaximin (Rifamycin class), and vancomycin (Glycopeptides class) (Shanghai Yuanye Biotechnology Co., Ltd., Shanghai, China) were determined against 49 *L. garvieae* isolates using micro-broth dilution assays, following the Clinical Laboratory and Standards Institute guidelines [42]. All antimicrobial agents were used in concentrations ranging from 0.03 to 16μg/mL. *S. aureus* ATCC 29,213 was used as a quality control strain. Antimicrobial resistance was defined by combining intermediate and resistant categories into a single category. MDR was defined as resistance to ≥3 classes of antimicrobial agents [2]. Each experiment was performed in triplicate.

### 2.8. Virulence Gene Detection

The following potential virulence genes were detected by PCR, with primers listed in Table 1, for 49 *L. garvieae* isolates: hly1 [34], hly2 [34], Hemolysin 3 (hly3) [34], NADHO [34], SOD [34], Phosphoglucomutase (pgm) [34], Pav [34], PsaA [34], eno [34], containing surface proteins-1, -2, -3, -4 (LP1, LP2, LP3, LP4) [34], AC1 [34], AC2 [34], Adhesin (Adh) [34], capsule gene cluster (1020-F, 1323-R) [34], capsule gene cluster (851-F, 1399-R) [34], capsule gene cluster (6329-F, 7175-R) [34], capsule gene cluster (5358-F, 6007-R) [34], conserved hypothetical protein (CHP) [34], exopolysaccharide R, X, A, B, C, D, and L (epsRXABCDL) [34], oligosaccharide repeat unit polymerase (ORUP) [37], rhamnosyltransferase (RIF) [37], and 30S rRNA gene [37]. The reaction mixtures (25 μL) consisted of 12.5 μL of Green Teq Mix, 1 μL of template DNA, 1 μL of each primer, and 9.5 μL of ultra-pure distilled water. Initial denaturation at 95 °C for 5 min was followed by 34 cycles of amplification at 95 °C for 15 s, annealing at 52 °C for 30 s (all primers), extension at 72 °C for 60 s, and a final extension step at 72 °C for 5 min. The PCR products were analyzed on 1.2% agarose gel. For the potential virulence genes, we confirmed that if an amplicon of the same size as that reported was observed, the isolate contained the target gene [34,37].

### 2.9. Cell Cultures

Bovine mammary alveolar cell T (MAC-T) (Shanghai Baiye Biotechnology Center, Shanghai, China) was prepared as previously described and cultured in Dulbecco’s Modified Eagle’s Medium (DMEM) supplemented with 10% fetal bovine serum (FBS) and 1% penicillin and streptomycin (Sigma Aldrich, St. Louis, MO, USA), at 37 °C with 5% CO_2_. 

### 2.10. Cytotoxic Lactate Dehydrogenase (LDH) Release Assay

LDH release was used to identify the most and least cytotoxic isolates among the 49 isolates for further pathogenicity studies, as well as to study the cytotoxic effects of *L. garvieae* on bovine mammary alveolar cell T (MAC-T) (Shanghai Baiye Biotechnology Center, Shanghai, China). This was assessed using an LDH assay kit (Beyotime Biotechnology, Beijing, China). Cells were cultured at 37 °C with 5% CO_2_ in 96-well plates (Corning Inc., Corning, NY, USA) and confluent growth (approximately 80% full) was achieved, then challenged with different *L. garvieae* isolates (n = 49) at a multiplicity of infection (MOI, ratio of *L. garvieae* to cells) of 5:1 for 12 h. The most cytotoxic isolate should have the highest LDH release, and the least cytotoxic should have the lowest LDH release; thus, the most and least cytotoxic isolates were selected for mouse mastitis model experiments. Then, MAC-T cells were cultured again at 37 °C with 5% CO_2_ in 96-well plates and challenged with the most and least cytotoxic isolates with an MOI of 5:1 at 1, 3, 6, 12, and 24 h. Uninfected cells were cultured as the control group. After incubation, 200 μL of the supernatant was collected from each well and transferred to a centrifuge tube and centrifuged (8000× *g*, 5 min at 4 °C). Then, 120 μL of the supernatant was transferred to a new 96-well polystyrene plate and 60 μL of reaction mixture was added to each well. The reaction mixture was then incubated in the dark on a rotating shaker (150 rpm) at room temperature for 30 min. The absorbance was read at 490 nm (QuantStudio3, Thermo Fisher, Waltham, MA, USA). Each experiment was performed in triplicate.

### 2.11. Adhesion Assay

To assess the adhesion capacity of *L. garvieae* to MAC-T, the bacterial adhesion of two *L. garvieae* isolates (LG41 and LG47) was slightly modified as described [2]. The MAC-T were cultured in 6-well plates (Corning Inc.) and confluent growth (approximately 80% full) was achieved in an antibiotic-free medium, followed by infection with *L. garvieae* at an MOI of 50:1 for 30 min, 1, 2, and 3 h, and cultured at 37 °C and 5% CO_2_. After incubation, cells were washed twice with sterile PBS (pH 7.4) to remove unbound bacteria. Adhered bacteria were released by adding 1 mL of PBS and 1 mL of 1% triton X-100 (0.5% vol/vol) to lyse cells. Both the bacterial suspension (1 mL) and cells in the control group were treated with 1 mL triton X-100. The cell lysates and treated bacterial supernatant of the infected group and bacterial supernatant of the control group were diluted using a 10-fold serial method, cultured on SBA, incubated at 37 °C for 24 h, and CFU counts were determined. The adhesion fraction was determined as follows:

$$\mathrm{Bacterial}\;\mathrm{adhesion}\;\mathrm{fraction}=\frac{\mathrm{bacterial}\;\mathrm{CFU}\;\mathrm{count}\;\mathrm{of}\;\mathrm{Cell}\;\mathrm{lysates}\;\mathrm{of}\;\mathrm{infected}\;\mathrm{group}\left(\mathrm{CFU}/\mathrm{mL}\right)}{\mathrm{bacterial}\;\mathrm{CFU}\;\mathrm{count}\;\mathrm{of}\;\mathrm{control}\;\mathrm{group}\;\left(\mathrm{CFU}/\mathrm{mL}\right)\;}\times 100\%$$

The adhesion assays were repeated 3 times, in triplicate for each test.

### 2.12. Morphology of Lactococcus garvieae on MAC-T

Gram staining and SEM of cell slides were carried out in order to observe the morphology of the co-culture of MAC-T and the two *L. garvieae* isolates (LG41 and LG47). The MAC-T were cultured in 6-well plates (Corning Inc.) for 2 days and grown to confluence in an antibiotic-free medium, followed by infection with *L. garvieae* (MOI 50:1) for 24 h. After incubation, cells were washed twice with sterile PBS (pH 7.4) to remove unbound bacteria. Gram staining was performed with stained with hematoxylin-eosin. After co-culturing for 24 h, an electron microscope fixing solution was added rapidly to fix the sample at room temperature for 2 h. Then, the samples were rinsed 3 times with 0.1 M phosphate buffer Pb (pH 7.4) for 15 min each time, and the samples were fixed with 1% osmic acid with 0.1 M phosphate buffer Pb (pH 7.4) at room temperature in the dark for 1–2 h. Afterwards, they were rinsed 3 times with 0.1 M phosphate buffer Pb (pH 7.4) for 15 min each time. After that, 30%–50%–70%–80%–90%–95%–100%–100% ethanol was injected into the tissue for 15 min each time. The final process of dehydration involved adding isoamyl acetate for 15 min; then, the sample was put into the critical point dryer for drying. The dried sample was placed on the sample table of the ion sputtering instrument and sprayed with gold for approximately 30 s. Finally, a scanning electron microscope was used to observe and collect pictures.

### 2.13. Lactococcus garvieae Experimental Infection in a Mouse Mammary Gland

**Figure 1:**
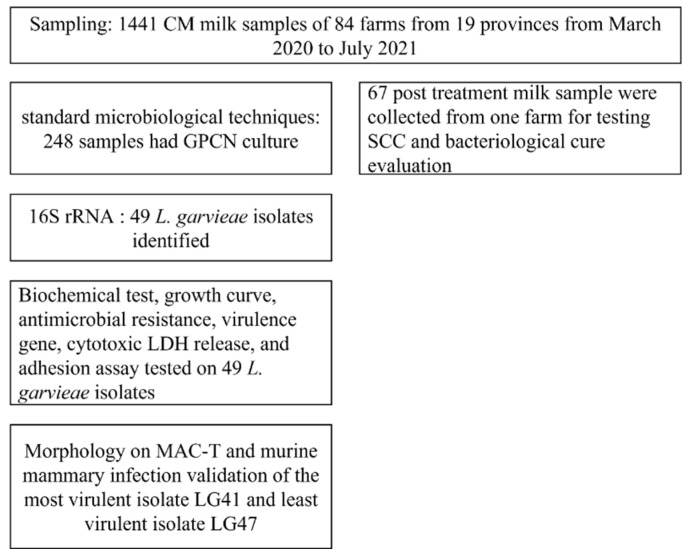
Flow diagram from sample collection and identification in the in vivo and in vitro study.

The pathogenic effect of two *L. garvieae* isolates (LG41 and LG47) during intramammary infection was determined using 6–8 week old female specific-pathogen-free BALB/c mice (Liaoning Changsheng Biotechnology Co., Ltd., Benxi, China) [43]. Pregnant (19 d of gestation) mice were kept in germ-free isolators and fed ad libitum in a controlled environment with light and dark cycles (12 h light and 12 h darkness). On the third day after parturition, mice were anesthetized by intramuscular injection of 50 mg/kg Zoletil 50 (Virbac, Carros, France). The fourth pair of mammary glands ducts were exposed by cutting the teat tip and 50 μL of bacterial suspension (5 × 107 CFU) was slowly injected using a small-gauge blunt-tipped needle (Guangdong Xiapute Technology Co., Ltd., Yangjiang, China). Three groups (n = 25 per group) of mice were allocated as 2 challenge groups (LG41 and LG47, respectively) and 1 negative control group (sterile PBS). The pups were removed 1 h before intramammary inoculation. The sedated mice were euthanized with cervical dislocation. The skin was fixed using pins before photographing the mammary glands. The bacterial load in the mammary glands at 12, 24, 48, 72, and 120 h after challenge (5 mice per time point) was measured as described [43]. Briefly, mammary gland tissue (0.1 g) was separated into a sterile Petri dish under a germ-free environment. After homogenization, 50 μL of supernatant was spread on sheep blood plates (multiple dilutions). The numbers of viable colonies were expressed as CFU/g. Mammary gland tissue was fixed with 5% paraformaldehyde, and embedded, sectioned, and stained with hematoxylin-eosin. Histological evaluation was performed to assess tissue necrosis, polymorphonuclear neutrophilic granulocyte inflammation (i.e., neutrophilic inflammation), and lymphocytic inflammation, as described [43]. The flow diagram showing the sample collection and identification for enrollment in the in vivo and in vitro study are shown in Figure 1.

### 2.14. Statistical Analyses

SPSS 22.0 (SPSS Corporation, Chicago, IL, USA) was used to analyze the data. One-way analysis of variance (ANOVA) was used to compare post-treatment SCC between *L. garvieae* and *L. lactis*, or other pathogen infected CM milk samples, LDH release, invasion and adhesion fractions between the treatment groups, and the Duncan test was used to determine the difference. If *p* < 0.05, the difference was considered to be statistically significant. 

## 3. Results

### 3.1. Morphological Characteristics of Lactococcus garvieae

**Figure 2:**
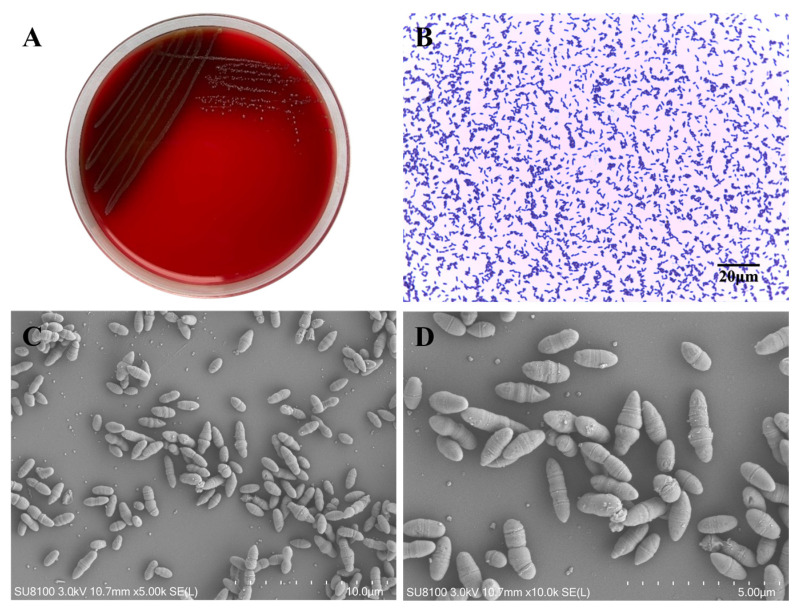
Morphological characteristics of *L. garvieae*. (**A**) Pinpoint colonies with α-hemolysis were observed on trypticase soy agar with 5% sheep blood; (**B**) Gram-positive cocci were observed under an optical microscope; and (**C**,**D**) SEM of *L. garvieae*.

*L. garvieae* formed round, medium-sized (approximately 1–2 mm in diameter) colonies on sheep blood TSA plates, with smooth edges, moist surfaces, and α light green hemolysis around the colony. The colony morphology and hemolysis was very similar to streptococcus after incubation at 37 °C for 24 h (Figure 2A). The bacteria stained gram-positive, and, microscopically, the bacterial body appeared spherical or ellipsoidal. The arrangement shape was either two to three bacteria lined up in short chains, or a single bacterium was present (Figure 2B). The results of the scanning electron microscopy showed that the bacteria were ellipsoidal in shape and approximately 1.5–2 μm in diameter. (Figure 2C,D). 

### 3.2. Identification of Suspected Isolates by 16S rDNA Sequence

After the initial isolation and identification of the 1441 milk samples, GPCN cocci isolates were subjected to 16S rDNA gene sequencing. A total of 248 GPCN cocci isolates (all suspected) were sequenced with 16S rDNA gene fragment amplicons, and the three bacteria with the highest percentage were *L. garvieae* (19.76%), *L. lactis* (16.53%), and *Streptococcus agalactiae* (13.71%). The details are shown in Table 2. Positive samples of *L. garvieae* were isolated from nine provinces. A total of 49 strains of *L. garvieae* were isolated and identified on 13 farms, with a sample detection frequency of 3.40% (49/1441). Between farms, there was a positive detection frequency of 16.25% (13/84). A farm in Ningxia had the highest detection frequency, with 31 strains of bacteria isolated from 149 clinical mastitis milk samples, and a detection frequency of 20.81%. The distribution of *L. garvieae* isolated from different Chinese commercial dairy farms are listed in Supplementary Materials in Table S1.

### 3.3. Biochemical Testing of Lactococcus garvieae

Biochemical testing of 49 *L. garvieae* isolates showed all negative results of substrate fermentation assays for ribose, sucrose, liquid gelatin, and sorbitol; 95.92% (47/49) positive results for lactose, maltose, trehalose, and glucose; 81.63% (40/49) positive results for galactose; and 79.59% (39/49) positive results for hesperidin. The VP test showed 77.55% (38/49) positive results, see Table 3. Detailed biochemical testing results are included in the Supplementary Materials.

### 3.4. Post-Treatment SCC and Bacteriological Cure

**Figure 3:**
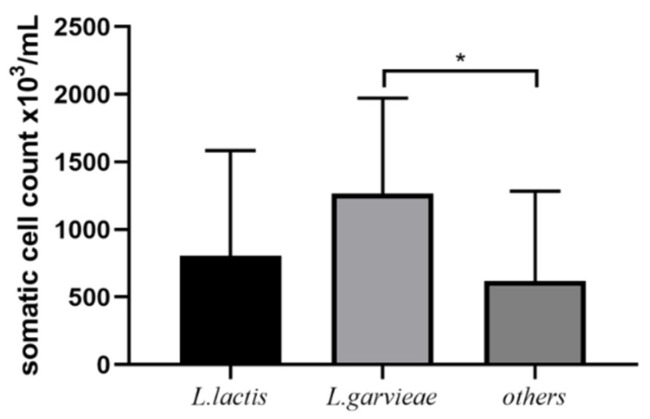
Post-treatment SCC of *L. lactis* vs. *L. garvieae* or others (includes *L. lactis*). * indicates a significant difference between different groups (*p* < 0.05 by ANOVA test).

Post-treatment SCC of *L. garvieae* (31 isolates), *L. lactis* (7 isolates), and other bacteria (7 *L. lactis* isolates, 2 *Aerococcus viridans* isolates, 1 *Enterococcus faecium* isolate, and 1 *S. uberis* isolate) are shown in Figure 3. Post-treatment SCC of *L. garvieae* infections was not significantly different to *L. lactis* but was significantly different from other bacterial isolates. The bacteriological cure fraction was 41.94% (13/31) for *L. garvieae*, 71.43% (5/7) for *L. lactis*, and 54.55% (6/11) for other bacteria (detailed bacteriological cure data not shown). There was no significant difference in the bacterial cure percentage, which is not surprising given the low numbers in the *L. lactis* and other groups. All 31 cows identified as being infected with mastitis caused by *L. garvieae* were classified as mild CM cases (abnormal milk only); however, eight had a recurrence within 30 days after initial diagnosis. The recurrence rate was 25.81% (8/31), while the recurrence rate of other pathogens was 9.10% (1/11).

### 3.5. Growth Ability of Lactococcus garvieae

**Figure 4:**
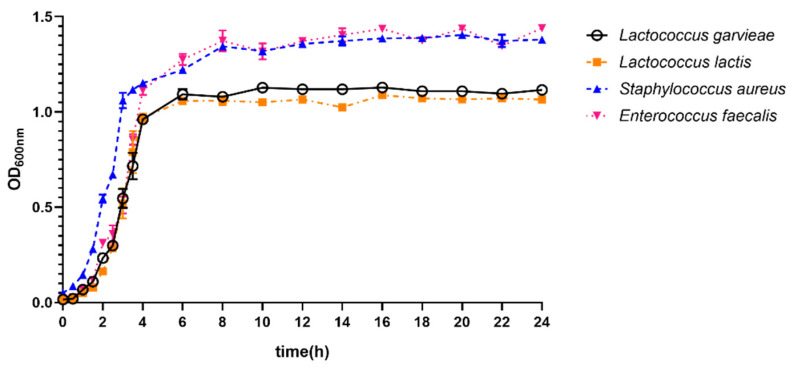
Growth curves of *L. garvieae*, *L. lactis*, *S. aureus*, and *E. faecalis* isolates. Data were mean ± SD of OD_600nm_ values of four isolates. The curves were plotted using GraphPad prism 8.

Growth curves of *L. garvieae*, *L. lactis*, *S. aureus*, and *E. faecalis* isolates cultured in BHI broth are shown in Figure 4. For *L. garvieae*, the bacterial growth curve consisted of a lag phase (~2 h), a log phase (~4 h), and ultimately, a stationary phase. Based on subjective observations, the growth curve of *L. garvieae* had a similar lag phase to other isolates. Additionally, the OD600nm value of *L. garvieae* isolates reached as high as ~1.0, whereas it was almost the same for *L. lactis* and up to ~1.3 for *S. aureus* and *E. faecalis* isolates. 

### 3.6. Antimicrobial Resistance Profiles of Lactococcus garvieae

All 49 *L. garvieae* isolates were susceptible to penicillin, ampicillin, ceftiofur, and cefquinome among the β-lactam antibiotics, but 12.24% were resistant to cephalexin. All *L. garvieae* isolates were resistant to lincomycin and rifaximin, and 73.47% of isolates were resistant to oxytetracycline. All *L. garvieae* isolates were sensitive to marbofloxacin and vancomycin, see Table 4. As *L. garvieae* is intrinsically resistant to clindamycin [44], it was excluded from the drug-resistant (DR) or multidrug-resistant (MDR) statistics in this paper. In summary, 89.20% (44/49) of *L. garvieae* were DR and 10.20% (5/49) were MDR. 

### 3.7. Detection of Virulence Genes in Lactococcus garvieae

**Figure 5:**
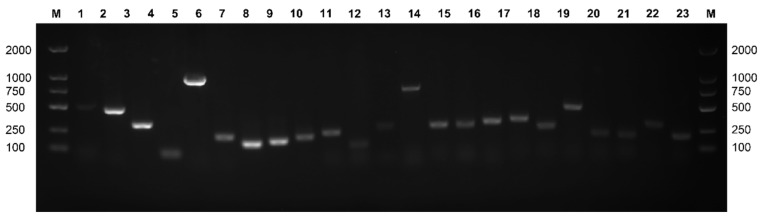
Identification of different virulence genes amongst *L. garvieae* isolates. M: 100 bp DNA ladder. Lanes 1−23: hly1, hly 2, NADHO, SOD, pgm, Pav, PsaA, eno, LP3, AC1, AC2, CGC (1020-F, 1323-R), CGC (6329-F, 7175-R), CHP, EpsA, EpsB, EpsC, EpsD, EpsL, EpsR, EpsX, RIF, and 30S rRNA gene.

Hemolysis-, adhesion-, and immune-evasion-related and other putative virulence genes of *L. garvieae* were examined. In total, two hemolysis genes (hly1 and hly2), five adhesion-related genes (Pav, PsaA, AC1, AC2, and LP3), nine immune-evasion-related genes (Capsule gene cluster (6329-F, 7175-R), capsule gene cluster (1020-F, 1323-R), EpsA, EpsB, EpsC, EpsD, EpsL, EpsR, and *EpsX*), and seven other putative virulence genes (NADHO, SOD, CHP, RIF, pgm, eno, and 30S gene) were detected. Of the five MDR *L. garvieae* isolates, hly2, Eps, and RIF were detected, while none of CGC-related genes were detected. Putative genes detected from the CM case are shown in Figure 5, and detailed putative virulence gene detect results are listed in Table 5.

### 3.8. Pathogenic Effects of Lactococcus garvieae on MAC-T 

**Figure 6:**
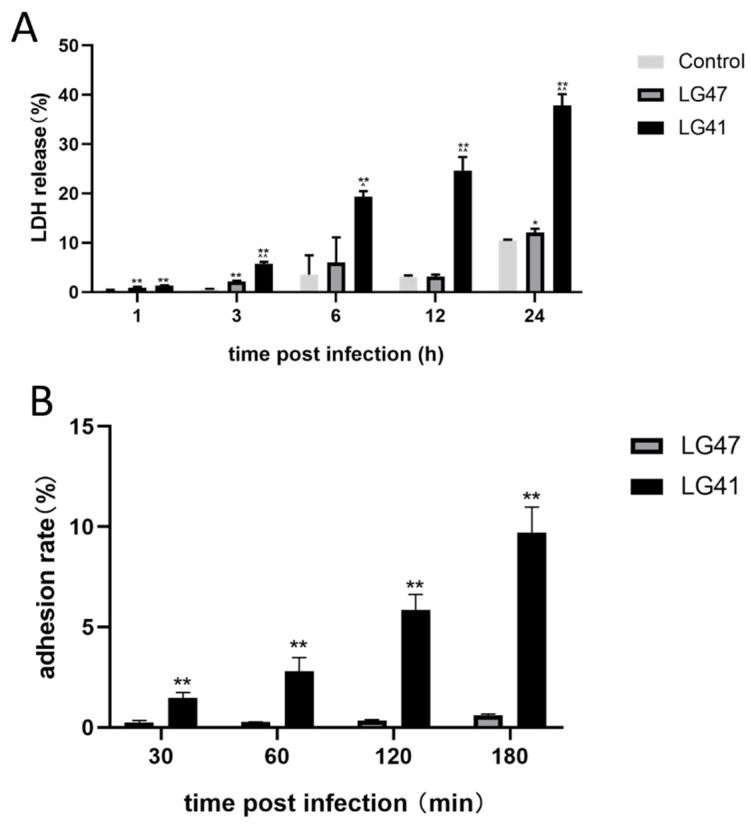
In vitro pathogenic effects of *L. garvieae* on bovine mammary epithelial cells (MAC-T). (**A**) Effects of *L. garvieae* isolates (LG41 and LG47) on lactate dehydrogenase release of MAC-T. Data are mean ± SD of three independent experiments. * indicates a significant difference between different treatment groups (*p* < 0.05 by ANOVA test), and ** indicates a very significant difference between different treatment groups (*p* < 0.01 by ANOVA test). (**B**) Adhesion of *L. garvieae* (up to 3 h after infection) into MAC-T. ** indicates a very significant difference between different treatment groups (*p* < 0.01 by ANOVA test). The bar charts were calculated and plotted using GraphPad prism 8.

LDH release 12 h after infection among different isolates indicated that LG41 was the most cytotoxic, while LG47 was the least cytotoxic. Therefore, these two isolates were selected. At 1, 3, 6, 12, and 24 h after infection, LDH release of LG41 was higher than in the control group (*p* < 0.01). At 1, 3, and 24 h after infection, LDH release of LG47 was higher than in the control group (*p* < 0.05). At 3, 6, 12, and 24 h after infection, LDH release of LG41 was higher than LG47 (*p* < 0.01, Figure 6A). The LG41 isolate adhered to MAC-T at a higher frequency compared with LG47 at the different time points (*p* < 0.01, Figure 6B).

### 3.9. Morphology of Lactococcus garvieae on MAC-T

**Figure 7:**
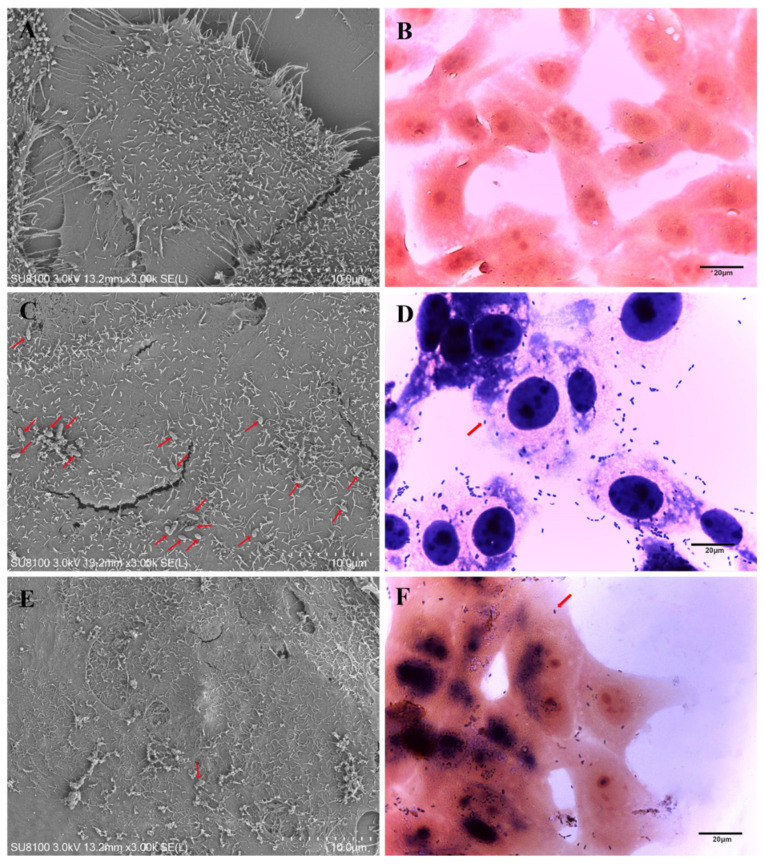
Image of morphology and structure of MAC-T observed by SEM, and the red arrow is bacteria. (**A**) Control group, (**C**) LG41 infection after 24 h, and (**E**) LG47 infection after 24 h. Gram staining image of morphology and structure of MAC-T; (**B**) control group, (**D**) LG41 infection after 24 h, and (**F**) LG47 infection after 24 h.

The cells in the control group were closely attached to the round coverslip, the cell morphology was paving stone-like, the surface of the cell membrane was covered with rich microvilli, the cells were arranged in an orderly manner, and there were elongated and rich pseudopods scattered in the cells, which was conducive to cell sticking (Figure 7A,B). After 24 h of LG41 challenge, the microvilli on the cell surface were broken, a large number of bacteria (red arrow) adhered to the cell surface, and there was damage to the cell surface where bacteria were attached. This phenomenon may be a manifestation of endocytosis (Figure 7C,D). After 24 h of LG47 challenge, there was a much smaller number of bacteria (red arrow) adhered to the cell surface than the LG41 group (Figure 7E,F).

### 3.10. Inflammation of Murine Mammary Gland Infected by Lactococcus garvieae

**Figure 8:**
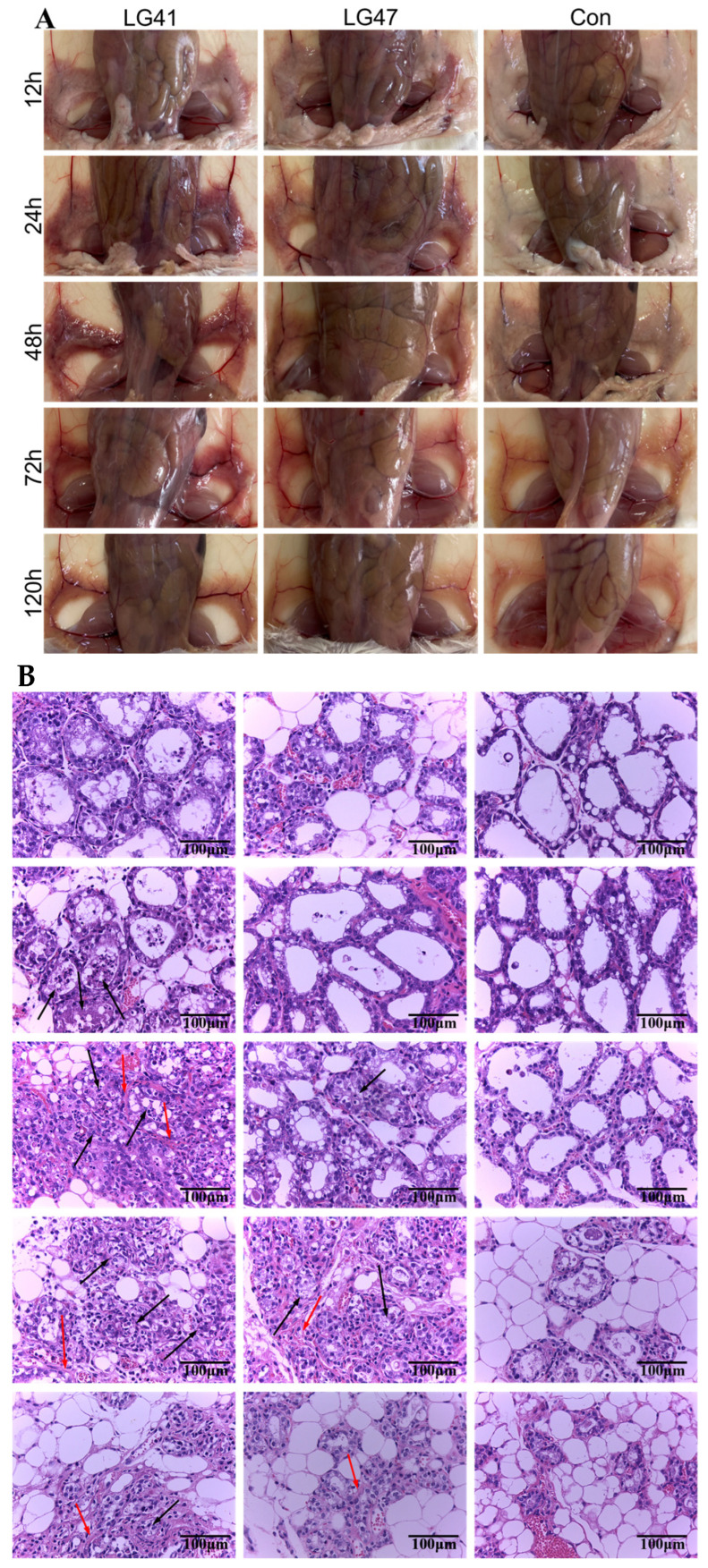

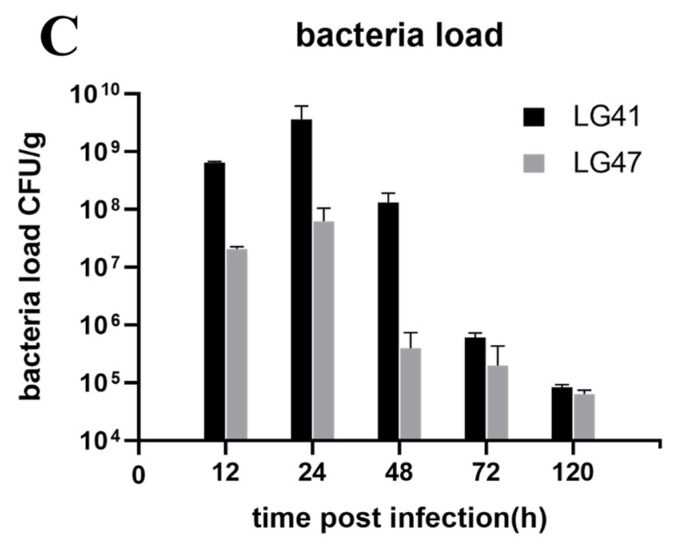
Pathological changes, histology, and bacterial load after mammary gland inoculation with *L. garvieae* in mice. (**A**) Pathological changes, including edema and hyperemia, in mammary glands of mice, challenged with two *L. garvieae* isolates (LG41 and LG47) and the control group. (**B**) Histological evaluation of mammary tissue (hematoxylin–eosin staining). *L. garvieae* provoked acute mastitis, with an increasing number of inflammatory cells (mainly neutrophils) infiltrated into the gland alveoli and interstitium of the mammary gland (black arrow) after infection, and connective tissue filled in the vacancy caused by inflammation after epithelial cell apoptosis (red arrow). CON = control. (**C**) Bacterial burden in mammary glands of mice (up to 120 h after inoculation). Each time point represents three mice in each of the five groups. There was no difference between *L. garvieae* isolates by ANOVA test. The bar charts were calculated and plotted using GraphPad prism 8.

Edema and hyperemia were evident in murine mammary glands at 12 h after infection with *L. garvieae*, with more profound pathological changes seen over time (24, 48, and 72 h after infection; Figure 8A). Histological characteristics of the mammary glands infected with *L. garvieae* were observed (Figure 8B). The structure of the gland alveoli was destroyed, the walls of the gland alveoli was thicker, and infiltrating inflammatory cells (mainly neutrophils) were observed in the gland alveoli and interstitium of the infected mammary gland at 24, 48, 72, and 120 h post infection. At 48 h post infection, the LG41 group showed interstitial tissue hyperplasia. Acute inflammation resolved at 120 h after infection, leaving connective tissue to fill the gap after epithelial death. No evidence of inflammation in mice from the control group (uninfected) was observed.

Bacteria were isolated from the mammary glands of mice challenged with *L. garvieae*, whereas no bacteria were isolated from the non-infected control group. The bacterial load (mean value) was 3.80 × 10^8^ CFU/g at 12 h after infection, but rapidly increased to 2.10 × 10^9^ CFU/g at 24 h after infection and started to drop to 7.55 × 10^7^ CFU/g at 48 h, 4.31 × 10^5^CFU/g at 72 h, and 7.49 × 10^4^ CFU/g at 120 h (Figure 8C).

## 4. Discussion

This is the first report of *L. garvieae* associated with bovine CM cases from multiple farms. *L. garvieae* is an emerging pathogen that has been confirmed with molecular testing methods such as PCR [5], RAPD, REP-PCR, MLRT, and MALDI-TOF [8]. The strain (98/4289) isolated from water was genetically more closely related to that from bovines than fish-oriented strains. This raises the possibility some environmental *L. garvieae* strains having evolved from mammals, and this may be involved in the epidemiology of fish lactococcosis [20]. This highlights the importance of implementing screening for *L. garvieae* as an emerging zoonotic bacterium. Humans, particularly those who have an anatomically or physiologically altered gastrointestinal tract or coexisting local predisposing health problems, are considered to be at-risk individuals. Some human cases have been associated with consuming raw seafood [8]. Consuming raw milk, therefore, has the potential for serious morbidity and mortality [10].

In the present study, the farms suffering from *L. garvieae* infection were using sand bedding. Sand bedding can be a reservoir of *L. garvieae* strains and be a potential vehicle for their dissemination in dairy farms. Contaminated sand bedding could also transfer infection between cows [21]. When *L. garvieae* was initially identified as causing bovine mastitis outbreaks on farms, it is possible the *Lactococcus* strain already existed and changes in the environment selectively favored the strain responsible for the outbreak. Alternatively, a new *Lactococcus* strain could have been introduced. *Lactococcus* has been found in samples from mastitic and normal milk, the bulk tank, and sand bedding. The relative abundance of the *Lactococcus* genus would be higher in the microbiome of mastitic samples, compared with milk samples from healthy animals [33]. 

The results of the MIC tests performed in this study agree with other studies that found *L. garvieae* to be resistant to clindamycin [8,47]. All isolates were sensitive to penicillin, ampicillin, ceftiofur, and cefquinome. However, 12.24% of isolates were resistant to cephalexin; this may be slightly biased in that the majority of isolates were from one farm that also had the most cytotoxic isolate. The IMM antibiotic tube used by the farm at the time of the study was a combination of cefalexin and kanamycin (Ubrolexin). Using a breakpoint of 16/1.6, as used by Sorge et al. (2021), 14.9% of the *L. garvieae* isolates were resistant to the cefalexin-kanamycin combination. In contrast, *L. garvieae* had a 5.3% resistance rate to marbofloxacin [48], but in this study, all isolates were sensitive; furthermore, all isolates were resistant to rifaximin. Rifaximin has frequently been used as an antibiotic in dry cow intramammary tubes on some Chinese dairy farms. It concerns us that, if a *Lactococcus* IMI outbreak occurred, rifaximin might not cure the subclinical infection in dry cows. Therefore, during the next lactation, these infected cows might be more likely to have a flare-up of a clinical case of *Lactococcus* mastitis. These cows would also be more likely to have a high individual SCC. The results of the MIC tests performed in this study also agree with other studies that *L. garvieae* from fish-derived [49] and from bovine-milk-derived [50] sources is resistance to tetracycline, and tetS genes were detected from all isolates. Of the 31 milk-derived *L. garvieae* isolates studied by Walther in 2008, 45.2% were resistant to tetracycline [50]. In comparison, the resistant fraction was 73.47% in this study. The MDR fraction of *L. garvieae* in this study was 10.20%, which was lower than other mastitic-milk-derived pathogenic bacteria reported in China, including 33% for *S. aureus*, 56% for non-aureus staphylococci, and 21% for *Streptococcus species* [51].

Previous reports have agreed that SCC will decline with time when a bacteriological cure is achieved, and this measure is a practical and reliable indicator of treatment success [1]. *Lactococcus* genus showed a lower bacteriological cure fraction and slower individual SCC resolution than *Streptococcus dysaglactiae* or *S. uberis* [52]. The bacteriological cure fraction of *L. garvieae* was comparable to that of *S. aureus*, ranging from 38.8% to 52% [53]. The bacteriological cure fraction of other GPCN bacteria isolated in this study (*L. lactis*, *Aerococcus viridans*, *Enterococcus faecium*, and *S. uberis*) were higher, suggesting that *L. garvieae* may be comparable to the major pathogenic bacteria in terms of the bacteriological cure fraction. On the other hand, the bacteriological cure fraction of *L. lactis* was significantly higher, suggesting that *L. lactis* might be less pathogenic than other pathogens. 

Extended therapy with ceftiofur was reported to provide a greater probability of bacteriological cure for gram-positive pathogens, both in clinical and subclinical cases [54,55]. Based on these results from previous studies and the MIC result of our study, we inferred that a low bacteriological cure might be improved by extended therapy. Control measures could also include vaccines, autovaccines, bacteriophages, and antiserum [7]. In bovines, an effective dry cow antibiotic tube could also be a good control method [56].

Furthermore, hly-1 was detected in all isolates, and hly-2 was detected in 97.96% of isolates. This was demonstrated by α-hemolysis of the bacterial colonies, as shown in Figure 2. The three adhesion genes (PavA, eno, and PsaA) were detected in all isolates [34]. Pgm is a metabolic enzyme conferring resistance to peptide antimicrobials and was detected in the most cytotoxic isolate; however, it was not detected in the least cytotoxic isolate. In this study, we investigated the presence of four LPxTG genes, and the LP3 gene was detected in 22.45% of the isolates; however, the LP1, LP2, and LP4 genes were not detected in any isolates. CGC was detected from fish-derived *L. garvieae*, and four different primers were used to detect *CGC* [37]; however, none of the isolates from CM cases had all four target stripes, which suggests the similarity between fish and bovines is not high. Seven genes (epsRXABCD) were conserved in the exopolysaccharide (EPS) biosynthetic gene cluster of 49 *L. garvieae* isolates. Similarly, these data suggest that capsule gene clusters may have spread wildly in *Lactococcus* spp. as genomic islands. The seven genes also appeared to encode enzymes involved in the polysaccharide structure of the capsule. It has been known that having only a few virulence genes is not enough to cause a pathogenic state, and an appropriate combination of virulence genes must be obtained to cause disease in a particular host species. The most cytotoxic isolate had more virulent genes identified than the least cytotoxic isolate. We inferred the difference in phenotypic virulence might be related to genotypic virulence, but further studies need to be carried out to confirm this.

In vivo challenge models in mice with bovine mastitis pathogens have been successfully used to assess bacterial infection and tissue damage [43]. In the current study, *L. garvieae* infections stimulated the inflammatory response of the murine mammary gland, which was manifested by the general appearance of concentrated inflammatory cell infiltration, progressive mammary alveolar damage, and the concentration of bacteria in the tissue. *L. garvieae* multiplied rapidly, leading to the migration of inflammatory cells to the mammary tissue, resulting in edema and congestion [2]. In addition to rapid growth in vitro and a high bacterial count, *L. garvieae* then declined but was not cleared. Infection with *L. garvieae* in the murine model indicated that the organism is well adapted to proliferation in the mammary gland and to cause tissue damage. The in vitro study (MAC-T) also supported the bacteria having good adaptive ability in bovine mammary cells.

## 5. Conclusions

This was the first time the zoonotic pathogen *L. garvieae* was isolated in CM milk samples from large dairy farms in China (prevalence of 3.40%). All *L. garvieae* isolates were susceptible to penicillin, ampicillin, cephalexin, cefquinome, ceftiofur, marbofloxacin, and vancomycin. *L. garvieae* had high resistance to lincomycin, oxytetracycline, and rifaximin, and 12.24% of isolates were resistant to cephalexin, with 10.20% (5/49) being multidrug-resistant (MDR). This suggests bacterial clearance may be decreased during the dry period after the application of dry cow antibiotic preparations and that extended therapy may result in better bacteriological cures in CM cases. The study demonstrated the adhesive ability of *L. garvieae* in MAC-T and how it can cause cell damage both in vitro and in vivo in the murine model of intramammary infection. The findings of this study help to explain the high prevalence, tissue-damaging nature, and antimicrobial resistance of *L. garvieae* as an emerging mastitis pathogen.

## Figures and Tables

**Table 1:** Primers of PCR.

Target Gene	Abbreviation of Target Gene	Primer Name	Primer Sequence (5′ to 3′)	Product Size
*Hemolysin 1*	*hly1*	H1 F	CCTCCTCCGACTAGGAACCA	521
		H1 R	GAAAAGCCAGCTTCTCGTGC	
*Hemolysin 2*	*hly2*	H2 F	TCTCGTGCACACCGATGAAA	492
		H2 R	TGAACTTCGGCTTCTGCGAT	
*Hemolysin 3*	*hly3*	H3 F	AACGCGAGAACAGGCAAAAC	291
		H3 R	CCCACGTCGAGAGCATAGAC	
*NADH oxidase*	*NADHO*	NADHO F	TGCGATGGGTTCAAGACCAA	331
		NADHO R	GCCTTTAAAAGCCTCGGCAG	
*Superoxide dismutase*	*SOD*	SOD F	GCAGCGATTGAAAAACACCCA	80
		SOD R	TCTTCTGGCAAACGGTCCAA	
*Phosphoglucomutase*	*pgm*	PG F	AAGTTTACGGCGAAGACGGT	997
		PG R	TTTTCTGGTGCATTGGCACG	
*Adhesin Pav*	*pav*	AP F	CCTGTCGGGCGCTTTTATTG	232
		AP R	TCCCGGAAGAAGAGTACGGT	
*Adhesin PsaA*	*PsaA*	APSA F	GTTGCAACAGCTGGACACAG	180
		APSA R	ATACGGTTGAGTTGGGCTGG	
*Enolase*	*eno*	E F	CAAGAGCGATCATTGCACGG	201
		E R	CATTCGGACGCGGTATGGTA	
*LPxTG-1*	*LP1*	LP1-F	GTGAACGTGGAGCTTCCAGA	878
		LP1-R	CCACTCACATGGGGGAGTTC	
*LPxTG-2*	*LP2*	LP2 F	GCCAGTGAGAGAACCGTTGA	767
		LP2 R	CAGGTTCAAGTGCAACTGCC	
*LPxTG-3*	*LP3*	LP3 F	TTAAGCACAACGGCAACAGC	231
		LP3 R	CACGCGAAATGATGGTGCAT	
*LPxTG-4*	*LP4*	LP4-F	GGGAGCACCGGATTCACTTT	928
		LP4-R	ACAAAGCCGCAGACCTTACA	
*Adhesin cluster 1*	*AC1*	AC1 F	TTGGGCACATCAGACTGGAC	264
		AC1 R	AGCATCATCAGCTGCCAAGT	
*Adhesin cluster 2*	*AC2*	AC2 F	CTGCGAGTGGCATCTCCATT	160
		AC2 R	TCAACACTGCGACCTTCTGT	
*Adhesin*	*AF*	AF F	CAGCCAGCACCAGGTTATGA	358
		AF R	CTCCTGCGTTGACATGGACT	
*capsule gene cluster A*	*CGC A*	1020-F	ACCTTCACTTGCATTCATAGGGT	304
		1323-R	TTGTCCCAGAGGGTTCTCCT	
*capsule gene cluster B*	*CGC B*	851-F	TAGGAGGTGTTCCTGGGAGG	549
		1399-R	TGTCCCACTCCTACTGTCGT	
*capsule gene cluster C*	*CGC C*	6329-F	AAAAACGGAGGGCAACAAGC	785
		7175-R	CACTTGTACAGGCCACTGGT	
*capsule gene cluster D*	*CGC D*	5358-F	TGGAGGGTATTGCCTACCGA	650
		6007-R	CCACAGCAGCTTCTTCACCT	
*conserved hypotherical protein*	*CHP*	CHP F	CTGCTGATCAAGTCCAAGC	303
		CHP R	GAGAAACGACCTTAGCTCCA	
*exopolysaccharide A*	*EpsA*	EpsA F	TTATAGCCTCCCCAGTTTACAC	299
		EpsA R	TTTAGCAGTCTCGTCTGCAATC	
*exopolysaccharide B*	*EpsB*	EpsB F	CGCAAGTGCTAATCTAGCTG	317
		EpsB R	AGAGAGGCGGAGTATCAATC	
*exopolysaccharide C*	*EpsC*	EpsC F	TAACAACTATCACTGCGACTCC	343
		EpsC R	TCAGGGTTCTCAATGATTCCAC	
*exopolysaccharide D*	*EpsD*	EpsD F	TTTCTTATTGCGGCTGCATTGC	270
		EpsD R	CTCATCAATTGAGTGTCGTCTG	
*exopolysaccharide L*	*EpsL*	EpsL F	ACCAATCGTACAGATCAACG	473
		EpsL R	CTTGAGCCACCACTATCAAG	
*exopolysaccharide R*	*EpsR*	EpsR F	TTTTACCACCGGCTAAAGGAAC	211
		EpsR R	TTGCAGAACTGTCATTAGGCTC	
*exopolysaccharide X*	*EpsX*	EpsX F	TATTGAAGCAACAGCCTCACTG	198
		EpsX R	TTTTTGTCTGGGTAACTAGCCC	
*rhamnosyltransferase*	*RIT*	RIT F	TTGATGGTAAATCCTGATGG	307
		RIT R	GAACAAACCGACCTACAACA	
*30S rRNA gene*	*30s rRNA gene*	30s F	TACGAACACCGTATCCTTGAC	207
		30s R	TTGTGTTGGTTCGATGATGTCG	

**Table 2:** Identification of suspected GPCN isolates by 16S rDNA sequence.

GPCN Cocci	Number of Isolates	Percent of Isolates ^a^ (%)
*Lactococcus garvieae*	49	19.76%
*Lactococcus lactis*	41	16.53%
*Streptococcus agalactiae*	34	13.71%
*Streptococcus dysgalactiae*	26	10.48%
*Streptococcus uberis*	25	10.08%
*Streptococcus lutetiensis*	20	8.06%
*Aerococcus viridans*	19	7.66%
*Enterococcus faecium*	15	6.05%
*Enterococcus faecalis*	13	5.24%
*Trueperella pyogenes*	6	2.42%
total	248	100.00%

^a^ = the number of specific pathogens/total number of all GPCN bacteria.

**Table 3:** Biochemical results of 49 *L. garvieae* isolates.

	Ribose	Sucrose	Lactose	Liquid Gelatin	Sorbitol	Maltose	Esculin	VP	Galactose	Trehalose	Glucose
Number of positives	0	0	47	0	0	47	39	38	40	47	47
Percentage positive	0.00%	0.00%	95.92%	0.00%	0.00%	95.92%	79.59%	77.55%	81.63%	95.92%	95.92%

**Table 4:** Number of isolates at each MIC value of antimicrobial agents against *L. garvieae*.

		MIC (μg/mL)													
Antimicrobial	Breakpoint	>16	16	8	4	2	1	0.5	0.25	0.12	0.06	0.03	Resistance Rate	MIC50 (μg/mL)	MIC90 (μg/mL)
Penicillin	16 ^a^							8	35	5	1		0.00%	0.25	0.5
Cephalexin	16 ^b^		6	28	13	1	1						12.24%	8	16
Ampicillin	8 ^a^							5	17	19	7		0.00%	0.12	0.5
Ceftiofur	2 ^c^					1	21	21		3	1		0.00%	0.5	1
Cefquinome	1 ^d^						1	3	15	29	1		0.00%	0.12	0.25
Lincomycin	4 ^e^		40	9									100.00%	16	16
Oxytetracycline	8 ^f^		12	4	20	8	4	1					73.47%	4	16
Marbofloxacin	8 ^g^						4	31	14				0.00%	0.5	0.5
Rifaximin	1 ^h^	49											100.00%	≥16	≥16
Vancomycin	32 ^i^							2	32	14	1		0.00%	0.25	0.25

^a^ = CLSI resistance breakpoint for enterococci [42]; ^b^ = CLSI resistance breakpoint of cephalothin for *Streptococcus* spp. [42]; ^c^ = CLSI resistance breakpoint for cattle mastitis [42]; ^d^ = breakpoint of cefquinome for *S. suis* [45]; ^e^ = CLSI resistance breakpoint of clindamycin for *Streptococcus* spp. [42]; ^f^ = CLSI breakpoints of tetracycline used for *Streptococcus* spp. [42]; ^g^ = CLSI resistance breakpoint for *Streptococcus* spp. [42]; ^h^ = Resistance breakpoint of rifampin [46]; ^i^ = CLSI breakpoints used in humans [42].

**Table 5:** Putative Virulence Gene Detection results of 49 *L. garvieae* isolates.

Genes	Number of Positives	Percentage Positive
*hly1*	49	100.00%
*hly2*	48	97.96%
*hly3*	0	0.00%
*NADHO*	49	100.00%
*SOD*	49	100.00%
*pgm*	15	30.61%
*Pav*	49	100.00%
*PsaA*	49	100.00%
*eno*	49	100.00%
*LP1*	0	0.00%
*LP2*	0	0.00%
*LP3*	11	22.45%
*LP4*	0	0.00%
*AC1*	49	100.00%
*AC2*	49	100.00%
*Adh*	0	0.00%
*1020-F, 1323-R*	2	4.08%
*851-F, 1399-R*	0	0.00%
*6329-F, 7175-R*	24	48.98%
*5358-F, 6007-R*	0	0.00%
*CHP*	44	89.80%
*EpsA*	49	100.00%
*EpsB*	46	93.88%
*EpsC*	49	100.00%
*EpsD*	35	71.43%
*EpsL*	42	85.71%
*EspR*	49	100.00%
*EspX*	49	100.00%
*ORUP*	0	0.00%
*RIF*	29	59.18%
*30S gene*	49	100.00%

## Data Availability

All of the relevant data are provided in the form of regular figures, tables, and Supplementary Materials Files.

## Supplementary Materials

The following supporting information can be downloaded at: https://www.mdpi.com/article/10.3390/microorganisms11020379/s1, Table S1: 1441 milk samples collected from 84 herds in 5 regions of China; Table S2: Biochemical results of 49 *L. garvieae* isolates; Table S3: Putative Virulence Gene Detection results of 49 *L. garvieae* isolates.
