# Supplementary material for: Prevalence, Virulence, and Antibiotics Gene Profiles in Lactococcus garvieae Isolated from Cows with Clinical Mastitis in China
Source: Microorganisms. 2023 Feb 2;11(2):379. doi: 10.3390/microorganisms11020379 (PMC9965093; doi:10.3390/microorganisms11020379)
Supplement: Supplementary file 1 [file microorganisms-11-00379-s001.zip › microorganisms-2152922-supplementary.pdf]

Table S1. 1441 milk samples collected from 84 herds in 5 regions of China.

region	Province confirmed with *L. garvieae*	Herd confirmed with *L. garvieae*	Samples, n	Samples with confirmed *L. garvieae*	
				By herd, n (%)	By region, %
Eastern China (5 provinces, 23 herds)			432		0.93
	Jiangsu	8th	17	2, (11.76%)	
	Jiangsu	9th	15	1, 6.67%	
	Shandong	21th	15	1, (6.67%)	
	Zhejiang	23th	13	1, (7.69%)	
Mid- China (3 provinces, 6 herds)			75		9.33
	Henan	25th	23	2, (8.70%)	
	Shanxi	28th	8	2, (25%)	
	Shanxi	29th	12	3, (25%)	
Northern China (6 provinces, 45 herds)			493		0.20
	Hebei	30th	17	1, (5.88%)	
Southern China (3 provinces, 8 herds)			83		2.41
	Yunnan	68th	11	2, (18.18%)	
Western China (2 provinces, 16 herds)			358		9.50
	Gansu	69th	17	1, (5.88%)	
	Ningxia	72th	67	31, (46.27%)	
	Ningxia	79th	26	1, (3.85%)	
	Ningxia	80th	9	1, (11.11%)	
Total			1441	49, (3.40%)	3.40

Table S2. Biochemical results of 49 *L. garvieae* isolates.

Isolates of *L. garvieae*	Ribose	sucrose	lactose	liquid gelatin	sorbitol	maltose	esculin,	VP	Galactose	trehalose	glucose
LG1	-	-	+	-	-	+	+	-	+	+	+
LG2	-	-	+	-	-	-	+	+	+	-	-
LG3	-	-	+	-	-	+	+	+	+	+	+
LG4	-	-	+	-	-	+	+	+	+	+	+
LG5	-	-	+	-	-	+	+	+	+	+	+
LG6	-	-	+	-	-	+	+	-	+	+	+
LG7	-	-	+	-	-	+	+	+	+	+	+
LG8	-	-	+	-	-	+	+	+	+	+	+
LG9	-	-	+	-	-	+	+	-	+	+	+
LG10	-	-	+	-	-	+	+	-	-	+	+
LG11	-	-	+	-	-	+	+	+	+	+	+
LG12	-	-	-	-	-	+	-	+	+	+	-
LG13	-	-	+	-	-	+	+	-	-	+	+
LG14	-	-	+	-	-	+	+	+	+	+	+
LG15	-	-	+	-	-	-	-	+	-	-	+
LG16	-	-	+	-	-	+	-	-	-	+	+
LG17	-	-	-	-	-	+	+	+	+	+	+
LG18	-	-	+	-	-	+	+	+	+	+	+
LG19	-	-	+	-	-	+	-	+	+	+	+
LG20	-	-	+	-	-	+	+	+	+	+	+
LG21	-	-	+	-	-	+	+	+	+	+	+
LG22	-	-	+	-	-	+	+	+	+	+	+
LG23	-	-	+	-	-	+	+	+	+	+	+
LG24	-	-	+	-	-	+	+	+	+	+	+

LG25	-	-	+	-	-	+	+	+	+	+	+
LG26	-	-	+	-	-	+	+	+	+	+	+
LG27	-	-	+	-	-	+	+	+	+	+	+
LG28	-	-	+	-	-	+	+	+	+	+	+
LG29	-	-	+	-	-	+	+	+	+	+	+
LG30	-	-	+	-	-	+	-	-	-	+	+
LG31	-	-	+	-	-	+	+	+	+	+	+
LG32	-	-	+	-	-	+	+	+	+	+	+
LG33	-	-	+	-	-	+	+	+	+	+	+
LG34	-	-	+	-	-	+	+	+	+	+	+
LG35	-	-	+	-	-	+	+	+	+	+	+
LG36	-	-	+	-	-	+	+	-	+	+	+
LG37	-	-	+	-	-	+	+	+	-	+	+
LG38	-	-	+	-	-	+	+	+	+	+	+
LG39	-	-	+	-	-	+	+	+	+	+	+
LG40	-	-	+	-	-	+	+	-	-	+	+
LG41	-	-	+	-	-	+	-	-	-	+	+
LG42	-	-	+	-	-	+	+	+	+	+	+
LG43	-	-	+	-	-	+	+	+	+	+	+
LG44	-	-	+	-	-	+	-	+	+	+	+
LG45	-	-	+	-	-	+	-	+	+	+	+
LG46	-	-	+	-	-	+	-	+	+	+	+
LG47	-	-	+	-	-	+	-	+	+	+	+
LG48	-	-	+	-	-	+	+	-	-	+	+
LG49	-	-	+	-	-	+	+	+	+	+	+

Table S3. Putative Virulence Gene Detection results of 49 *L. garvieae* isolates.

Isolates of L. garvieae	hly1	hly2	hly3	NADHO	SOD	pgm	Pav	PsaA	eno	LP1	LP2	LP3	LP4	AC1	AC2	Adh	1020-F, 1323-R	851-F, 1399-R	6329-F, 7175-R	5358-F, 6007-R	CHP	EpsA	EpsB	EpsC	EpsD	EpsL	EspR	EspX	ORUP	RIF	30S
LG1	+	+	-	+	+	+	+	+	+	-	-	-	-	+	+	-	-	-	+	-	+	+	+	+	-	+	+	+	-	-	+
LG2	+	+	-	+	+	+	+	+	+	-	-	-	-	+	+	-	-	-	+	-	+	+	+	+	+	+	+	+	-	+	+
LG3	+	+	-	+	+	+	+	+	+	-	-	-	-	+	+	-	-	-	-	-	-	+	+	+	-	+	+	+	-	+	+
LG4	+	+	-	+	+	-	+	+	+	-	-	-	-	+	+	-	-	-	-	-	-	+	+	+	-	+	+	+	-	-	+
LG5	+	+	-	+	+	-	+	+	+	-	-	-	-	+	+	-	-	-	+	-	+	+	+	+	-	+	+	+	-	-	+
LG6	+	+	-	+	+	-	+	+	+	-	-	-	-	+	+	-	-	-	-	-	+	+	-	+	-	+	+	+	-	-	+
LG7	+	+	-	+	+	-	+	+	+	-	-	-	-	+	+	-	-	-	-	-	+	+	+	+	-	+	+	+	-	+	+
LG8	+	+	-	+	+	-	+	+	+	-	-	-	-	+	+	-	-	-	+	-	+	+	-	+	-	+	+	+	-	-	+
LG9	+	+	-	+	+	-	+	+	+	-	-	-	-	+	+	-	-	-	-	-	+	+	-	+	-	+	+	+	-	-	+
LG10	+	+	-	+	+	-	+	+	+	-	-	-	-	+	+	-	-	-	-	-	+	+	+	+	-	-	+	+	-	-	+
LG11	+	+	-	+	+	-	+	+	+	-	-	-	-	+	+	-	-	-	-	-	+	+	+	+	-	-	+	+	-	+	+
LG12	+	+	-	+	+	-	+	+	+	-	-	-	-	+	+	-	-	-	+	-	+	+	+	+	-	+	+	+	-	+	+
LG13	+	+	-	+	+	-	+	+	+	-	-	-	-	+	+	-	-	-	-	-	+	+	+	+	-	+	+	+	-	+	+
LG14	+	+	-	+	+	-	+	+	+	-	-	+	-	+	+	-	+	-	+	-	+	+	+	+	+	+	+	+	-	-	+
LG15	+	+	-	+	+	-	+	+	+	-	-	-	-	+	+	-	-	-	-	-	+	+	+	+	+	+	+	+	-	-	+
LG16	+	+	-	+	+	+	+	+	+	-	-	-	-	+	+	-	-	-	+	-	+	+	+	+	+	+	+	+	-	-	+
LG17	+	+	-	+	+	+	+	+	+	-	-	-	-	+	+	-	-	-	+	-	+	+	+	+	+	+	+	+	-	+	+
LG18	+	+	-	+	+	+	+	+	+	-	-	-	-	+	+	-	-	-	-	-	+	+	+	+	+	+	+	+	-	+	+
LG19	+	+	-	+	+	-	+	+	+	-	-	-	-	+	+	-	-	-	-	-	+	+	+	+	+	-	+	+	-	+	+
LG20	+	+	-	+	+	-	+	+	+	-	-	-	-	+	+	-	-	-	-	-	+	+	+	+	+	+	+	+	-	+	+
LG21	+	+	-	+	+	-	+	+	+	-	-	-	-	+	+	-	-	-	-	-	+	+	+	+	+	+	+	+	-	-	+
LG22	+	+	-	+	+	+	+	+	+	-	-	-	-	+	+	-	-	-	-	-	+	+	+	+	+	+	+	+	-	-	+

LG23	+	+	-	+	+	+	+	+	+	-	-	-	-	+	+	-	-	-	-	-	+	+	+	+	+	+	+	+	-	-	+
LG24	+	+	-	+	+	+	+	+	+	-	-	-	-	+	+	-	-	-	-	-	+	+	+	+	+	+	+	+	-	-	+
LG25	+	+	-	+	+	-	+	+	+	-	-	-	-	+	+	-	-	-	+	-	-	+	+	+	+	+	+	+	-	+	+
LG26	+	+	-	+	+	-	+	+	+	-	-	-	-	+	+	-	-	-	-	-	+	+	+	+	+	+	+	+	-	-	+
LG27	+	+	-	+	+	-	+	+	+	-	-	+	-	+	+	-	-	-	-	-	+	+	+	+	+	+	+	+	-	+	+
LG28	+	+	-	+	+	-	+	+	+	-	-	+	-	+	+	-	-	-	-	-	+	+	+	+	+	+	+	+	-	+	+
LG29	+	+	-	+	+	+	+	+	+	-	-	-	-	+	+	-	-	-	+	-	+	+	+	+	+	+	+	+	-	-	+
LG30	+	+	-	+	+	-	+	+	+	-	-	-	-	+	+	-	-	-	+	-	+	+	+	+	+	+	+	+	-	+	+
LG31	+	+	-	+	+	+	+	+	+	-	-	+	-	+	+	-	-	-	+	-	+	+	+	+	+	+	+	+	-	+	+
LG32	+	+	-	+	+	+	+	+	+	-	-	+	-	+	+	-	-	-	+	-	+	+	+	+	+	+	+	+	-	+	+
LG33	+	+	-	+	+	+	+	+	+	-	-	+	-	+	+	-	-	-	+	-	+	+	+	+	+	+	+	+	-	-	+
LG34	+	+	-	+	+	-	+	+	+	-	-	+	-	+	+	-	-	-	+	-	+	+	+	+	+	+	+	+	-	-	+
LG35	+	+	-	+	+	-	+	+	+	-	-	+	-	+	+	-	-	-	+	-	+	+	+	+	+	+	+	+	-	-	+
LG36	+	+	-	+	+	-	+	+	+	-	-	-	-	+	+	-	-	-	-	-	+	+	+	+	-	+	+	+	-	-	+
LG37	+	+	-	+	+	-	+	+	+	-	-	-	-	+	+	-	-	-	+	-	+	+	+	+	+	+	+	+	-	+	+
LG38	+	+	-	+	+	-	+	+	+	-	-	+	-	+	+	-	-	-	+	-	+	+	+	+	+	+	+	+	-	+	+
LG39	+	+	-	+	+	-	+	+	+	-	-	+	-	+	+	-	-	-	+	-	+	+	+	+	+	-	+	+	-	+	+
LG40	+	+	-	+	+	-	+	+	+	-	-	-	-	+	+	-	-	-	-	-	-	+	+	+	+	-	+	+	-	+	+
LG41	+	+	-	+	+	+	+	+	+	-	-	-	-	+	+	-	+	-	+	-	-	+	+	+	+	+	+	+	-	+	+
LG42	+	-	-	+	+	-	+	+	+	-	-	-	-	+	+	-	-	-	+	-	+	+	+	+	+	+	+	+	-	+	+
LG43	+	+	-	+	+	-	+	+	+	-	-	-	-	+	+	-	-	-	-	-	+	+	+	+	-	+	+	+	-	+	+
LG44	+	+	-	+	+	-	+	+	+	-	-	+	-	+	+	-	-	-	-	-	+	+	+	+	+	+	+	+	-	+	+
LG45	+	+	-	+	+	-	+	+	+	-	-	-	-	+	+	-	-	-	-	-	+	+	+	+	+	-	+	+	-	+	+
LG46	+	+	-	+	+	-	+	+	+	-	-	-	-	+	+	-	-	-	+	-	+	+	+	+	+	+	+	+	-	+	+
LG47	+	+	-	+	+	-	+	+	+	-	-	-	-	+	+	-	-	-	-	-	+	+	+	+	+	+	+	+	-	+	+
LG48	+	+	-	+	+	-	+	+	+	-	-	-	-	+	+	-	-	-	+	-	+	+	+	+	+	-	+	+	-	+	+

LG49	+	+	-	+	+	+	+	+	+	-	-	-	-	+	+	-	-		-		+		-		+	+	+	+	+	+	+	+	-	+	+
